# CD229 interacts with RASAL3 to activate RAS/ERK pathway in multiple myeloma proliferation

**DOI:** 10.18632/aging.204405

**Published:** 2022-11-28

**Authors:** Zigen Lin, Xiaozhu Tang, Yuhao Cao, Lijin Yang, Mingmei Jiang, Xinying Li, Jie Min, Bing Chen, Ye Yang, Chunyan Gu

**Affiliations:** 1 Department of Hematology, Nanjing Drum Tower Hospital, The Affiliated Hospital of Nanjing University of Chinese Medicine, Nanjing, China; 2 School of Medicine and Holistic Integrative Medicine, Nanjing University of Chinese Medicine, Nanjing, China

**Keywords:** multiple myeloma, CD229, RAS, RASAL3

## Abstract

Multiple myeloma (MM) is an incurable plasma cell malignancy, while CAR-T therapy offers a new direction for the treatment of MM. Recently, signaling lymphocytic activation molecule family 3 (CD229), a cell surface immune receptor belonging to the signaling lymphocyte activating molecule family (SLAMF), is emerging as a CAR-T therapeutic target in MM. However, a clear role of CD229 in MM remains elusive. In this study, MM patients with elevated CD229 expression achieved poor prognosis by analyzing MM clinical databases. In addition, CD229 promoted MM cell proliferation *in vitro* as well as in xenograft mouse model *in vivo*. Mechanism study revealed that CD229 promoted MM cell proliferation by regulating the RAS/ERK signaling pathway. Further exploration employed co-immunoprecipitation coupled with mass spectrometry to identify RASAL3 as an important downstream protein of CD229. Additionally, we developed a co-culture method combined with the immunofluorescence assay to confirm that intercellular tyrosine phosphorylation mediated self-activation of CD229 to activate RAS/ERK signaling pathway via interacting with RASAL3. Taken together, these findings not only demonstrate the oncogenic role of CD229 in MM cell proliferation, but also illustrate the potential of CD229 as a promising therapeutic target for MM treatment.

## INTRODUCTION

Multiple myeloma (MM) is a hematological B-lymphocyte malignancy with clonal expansion of plasma cells in the patient's bone marrow, which can ultimately cause hypercalcemia, renal failure, anemia, bone disease and immunosuppression [1, 2]. MM patients are divided into 8 groups based on gene expression profiling (GEP) [3, 4], including CD1 and CD2 subgroup with spiked expression of CCND1 and CCND3, hyperdiploidy (HY) group, low bone disease (LB) group, MAF/MAFB (MF) spike group, MMSET spike (MS) group, myeloid-like (MY) group and proliferation (PR) group. PR group is considered as the highest-risk MM subgroup and the patients in PR group suffer from the worst prognosis [5]. Despite the fact that the great advancement of new therapies, such as proteasome inhibitors, has dramatically improved outcomes for MM patients over the past decades, almost all patients eventually experience inevitable MM relapse and die of this disease [6, 7].

It is of note that chimeric antigen receptor T (CAR-T) cell therapy has been already introduced for treating MM in clinical trials [8–11]. Recently, CAR-T cell therapy targeting B-cell maturation antigen (BCMA) has shown high-response rates, but the clinical application of CAR-T cell therapy is restricted mainly due to its limited durability [12]. CD229 is a potential target for CAR-T cell therapy owing to its homogenous expression in MM cells, and MM cells depend on CD229 for their survival [13]. As a member of the signaling lymphocytic activation molecule family (SLAMF), CD229 acts as a cell-surface immune receptor and is involved in immune response mediated by immune cells [14, 15]. Besides, CD229 is expressed in hematopoietic stem cells, T cells, B lymphocytes and NK cells [16]. Importantly, CD229 is homogeneously expressed in the bulk of malignant plasma cells from MM patients as well as chemotherapy-resistant myeloma progenitors [17–19]. The extracellular domain of CD229 is mainly composed of 4 immunoglobulin-like domains (two tandem repeats of IgV and IgC2). The cytoplasmic domain of CD229 contains two immunoreceptors tyrosine-based signaling motifs (ITSMs, T-V/I-Y-xx-V/I) and several tyrosine residues as SH2 domain binding sites following phosphorylation [20]. The intracellular motifs are critical for CD229 signaling that regulate ERK phosphorylation in T cell receptor (TCR) signaling pathway [21]. In addition, CD229 is associated with enhanced T cell activation and Th2 polarization [22, 23]. Hence, the surface molecule CD229 is expected to be a promising target for anti-MM immunotherapy. However, the critical role of CD229 in MM remains unclear.

Inspired by the current studies on the effective efficacy of CD229-based CAR-T therapy, we herein examined the expression of CD229 in a subpopulation of myeloma, and demonstrated the role of CD229 in promoting MM cell proliferation through the underlying mechanism of CD229-mediated RAS/ERK signaling. It is suggested that CD229 may be served as a novel biological target for treatment of MM.

## RESULTS

### Elevated CD229 expression confers poor survival in MM

**Figure 1:**
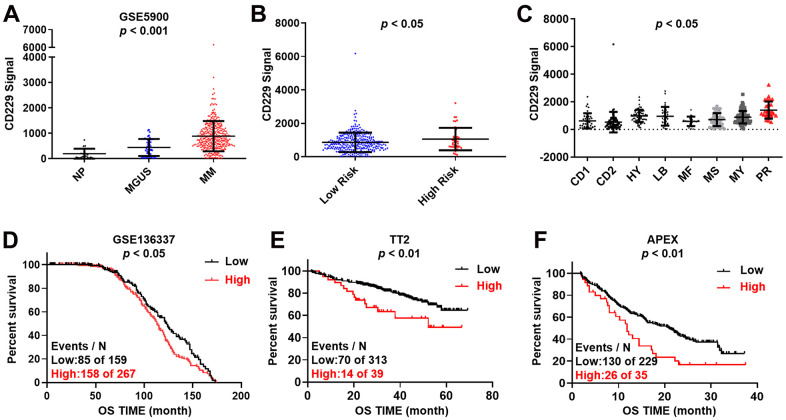
**Increased CD229 expression is correlated with poor survival in MM.** (**A**) CD229 mRNA levels were significantly elevated in MM patients. The signal level of CD229 was shown on the y-axis. Patients were designated as healthy donors with normal bone marrow plasma cells (NP, n = 22), monoclonal gammopathy of undetermined significance (MGUS, n = 44), or multiple myeloma (MM, n = 351), sorted on the x-axis. (**B**, **C**) Box plot showed CD229 expression in high-risk versus low-risk MM subgroup (**B**), and in 8 MM risk subgroups from TT2 patient cohort (**C**). (**D**–**F**) Elevated CD229 mRNA expression was associated with poor overall survival (OS) in MM patients from GSE136337 (**D**), TT2 (**E**) and APEX (**F**) patient cohorts.

To assess the expression of CD229 in MM, we first examined the expression of CD229 in normal plasma (NP, n = 22), monoclonal gammopathy of undetermined significance (MGUS, n = 44) and MM patients (MM, n = 351) (gene expression dataset GSE5900). The results showed that the CD229 mRNA level was significantly increased from NP, MGUS to MM patients during disease progression (*p* < 0.001) (Figure 1A). Intriguingly, we also observed higher CD229 expression in high-risk MM patients than low-risk patients in the GSE2658 dataset (*p* < 0.05) (Figure 1B). In detail, CD229 expression in PR group, the worst subgroup in MM patients, was dramatically higher than those in the other 7 subgroups (*p* < 0.05) (Figure 1C). High expression of CD229 was significantly associated with poor overall survival (OS) in GSE136337 (*p* < 0.05) (Figure 1D). Furthermore, MM patients bearing elevated CD229 expression suffered from poor prognosis in both newly diagnosed MM from TT2 (Total Therapy 2) cohort (*p* < 0.01) (Figure 1E) and relapsed MM patients from APEX (Assessment of Proteasome inhibition for Extending remissions) cohort (*p* < 0.01) (Figure 1F). Collectively, these results demonstrate that CD229 acting as an oncogene may confer a poor prognosis in MM patients.

### CD229 promotes MM cell proliferation

**Figure 2:**
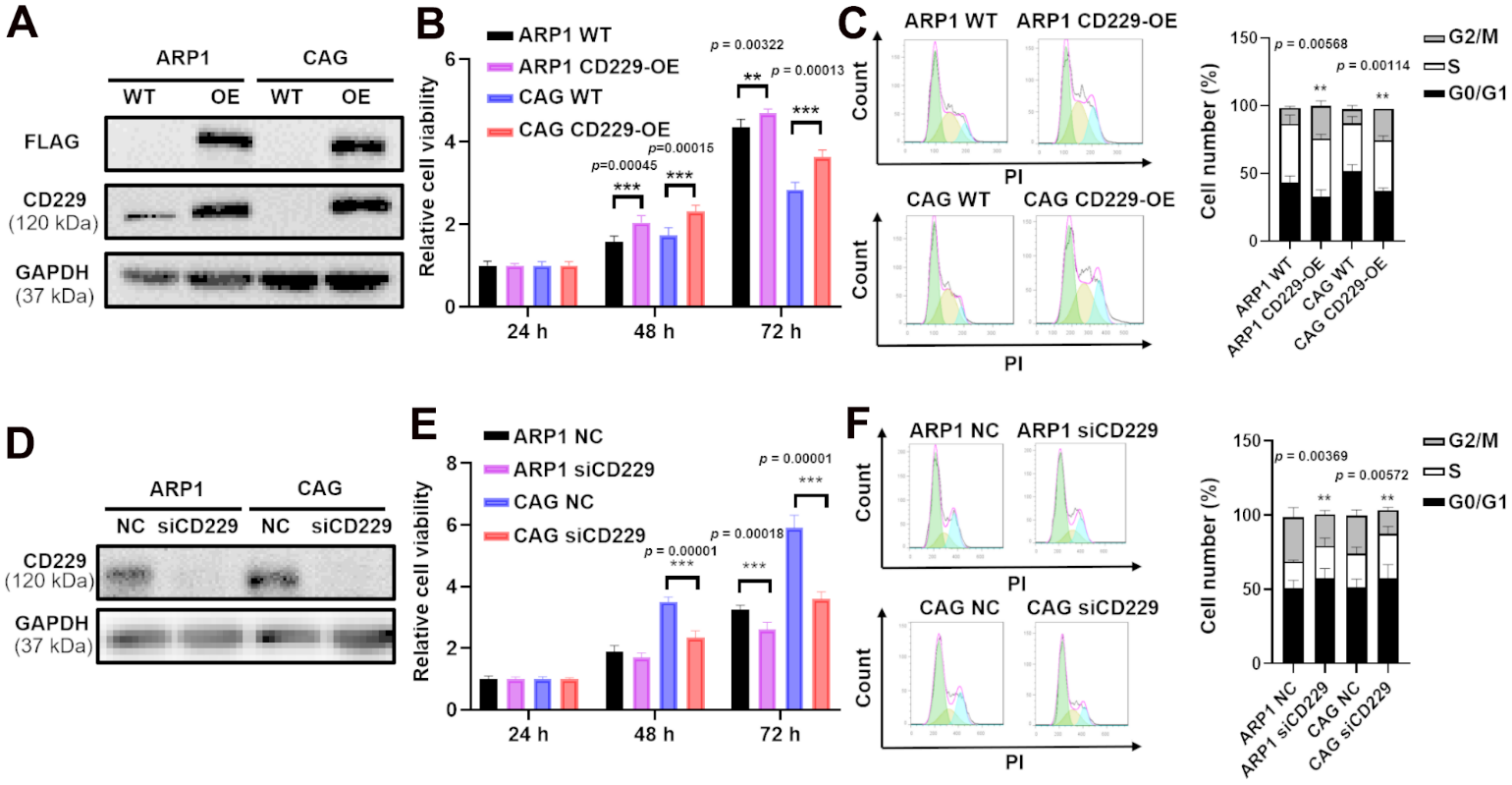
**Elevated CD229 expression promotes MM cell proliferation.** (**A**) WB analysis of CD229 overexpression in ARP1 and CAG cells using CD229 and Flag tag antibodies. (**B**) The proliferation capacity in WT and CD229-OE MM cells was detected by MTT. (**C**) Flow cytometry revealed that the proportion of G2/M phase was significantly increased in CD229-OE cells compared to WT cells. (**D**) WB analysis confirmed the reduction of CD229 in ARP1 and CAG cells upon transfection with the siRNA. (**E**) Decreased CD229 resulted in a lower cell proliferation rate in ARP1 and CAG cells detected by MTT. (**F**) Flow cytometry analysis revealed that the proportion of G2/M phase was significantly decreased in si-CD229 cells relative to NC cells. The data of experiments represent Mean±SD from at least three independent experiments. **p < 0.01, ***p < 0.001.

To determine whether CD229 could function as a contributing factor for MM cell proliferation, CD229 was overexpressed (OE) in MM cells through lentiviral transfection, as confirmed by Western blotting (WB) (Figure 2A and Supplementary Figure 1A, 1B). MTT assay showed that the proliferation of CD229-OE MM cells was significantly enhanced compared to wildtype (WT) cells (*p* < 0.01) (Figure 2B). Cell cycle analysis demonstrated that the proportion of G2/M phase in CD229-OE MM cells was evidently higher than that in WT cells (*p* < 0.01) (Figure 2C). Inversely, CD229 was knocked down (KD) by CD229-targeting siRNA, as validated by WB (Figure 2D and Supplementary Figure 1C). CD229-KD MM cells displayed significantly lower cell growth rates than the negative control (NC) cells (*p* < 0.001) (Figure 2E). In addition, the cell cycle analysis showed an obviously lower proportion of G2/M phase in CD229-KD MM cells compared to NC cells (*p* < 0.01) (Figure 2F). Taken together, these findings further indicate that CD229 stimulates MM cell proliferation.

### RAS pathway participates in CD229-induced MM cell proliferation

**Figure 3:**
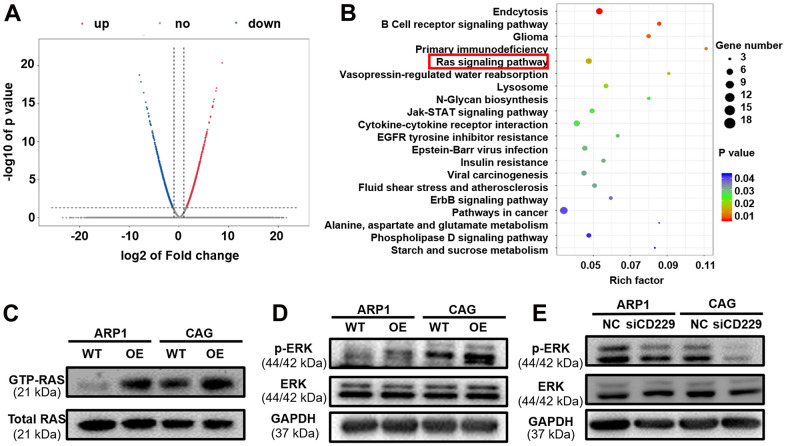
**The potential signaling pathway of CD229 for MM biology is screened by RNA-seq.** (**A**) The volcano plot of differentially expressed genes between WT and CD229-OE MM cells. X axis, log2 fold change; Y axis, −log10 P value. (**B**) Pathway enrichment analysis of RNA-seq data unveiled enrichment of RAS signaling pathway. (**C**) RAS-GTP and total RAS expression in WT and CD229-OE MM cells were detected by RAS antibody. (**D**, **E**) WB test confirmed that p-ERK was increased in CD229-OE cells (**D**) and decreased in si-CD229 cells (**E**). The data of experiments represent Mean±SD from at least three independent experiments.

To further explore the mechanism by which CD229 affected MM cell proliferation, we prepared RNA samples for transcriptomic RNA sequencing (RNA-seq) to screen differentially expressed genes between CD229-OE cells and their corresponding WT cells. In total, we found that 236 genes were upregulated and 254 genes were downregulated in CD229-OE MM cells compared to WT cells (Figure 3A). As shown in Figure 3B (see Supplementary Table 1 for statistics of top 30 enriched pathways), the KEGG pathway enrichment analysis elucidated that multiple signaling pathways were significantly enriched, such as Endocytosis, B Cell receptor signaling pathway, Primary immunodeficiency and RAS signaling pathway. Among them, the RAS signaling pathway was most closely related to tumor development. Then we examined the RAS-GTP as well as total RAS expression. Intriguingly, the results showed that RAS-GTP but not total RAS was increased in CD229-OE MM cells (Figure 3C and Supplementary Figure 1D, 1E). Furthermore, we examined total ERK and phosphorylated ERK (p-ERK) levels in WT and CD229-OE MM cells. The results showed that p-ERK levels were increased in CD229-OE MM cells and decreased in CD229-KD MM cells, while no obvious changes were observed in the expression of total ERK (Figure 3D, 3E and Supplementary Figure 1F–1I). The above data provide the evidence that elevated expression of CD229 can activate RAS/ERK pathway to induce MM cell proliferation.

### Overexpression of CD229 promotes tumor proliferation *in vivo*

**Figure 4:**
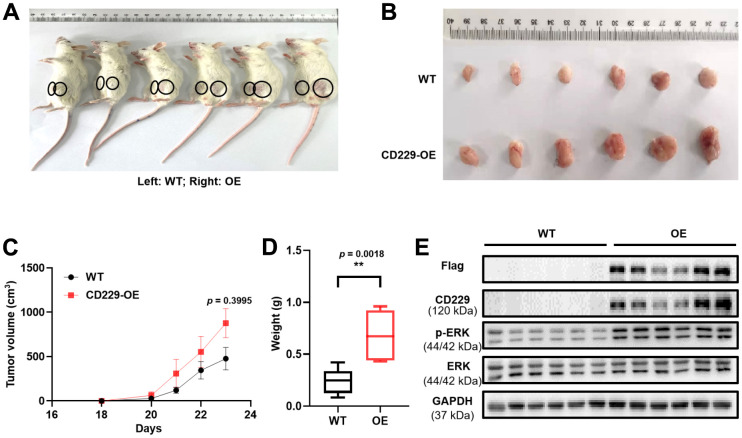
**Overexpression of CD229 promotes MM cell proliferation in MM xenograft model.** (**A**) Photographic images of xenograft mice were captured on Day 23. (**B**) Photographic images of xenografts from SCID/NOD mice. (**C**) Tumor volume growth curve of NOD/SCID mice. (**D**) Tumor weight in CD229-OE group was significantly higher than those of WT group. (**E**) WB assay showed that both CD229 and p-ERK expression were higher in CD229-OE group than WT group derived from xenograft tumors. The data of experiments represent Mean±SD from at least three independent experiments. **p < 0.01.

As to confirm the oncogenic role of CD229 *in vivo*, the xenograft mouse model was constructed in the immunodeficient NOD/SCID mice by subcutaneous injection of WT and CD229-OE MM cells into the left and right flank of mice, respectively. Interestingly, we observed that the volume of the abdominal tumor formed by CD229-OE MM cells appeared larger than those formed by WT MM cells (Figure 4A–4C). Similarly, the tumor weight in CD229-OE group was significantly higher than that in WT group (*p* < 0.01) (Figure 4D). To further verify the activation of RAS/ERK pathway by CD229 *in vivo*, the CD229 and p-ERK expressions of the excised tumor were detected by WB assays. The *in vivo* results showed that CD229 was stably overexpressed and p-ERK was activated in CD229-OE group compared to WT group (Figure 4E), suggesting a critical role of CD229 on RAS/ERK pathway.

### CD229 interacts with RASAL3 protein to regulate the RAS/ERK pathway in MM

**Figure 5:**
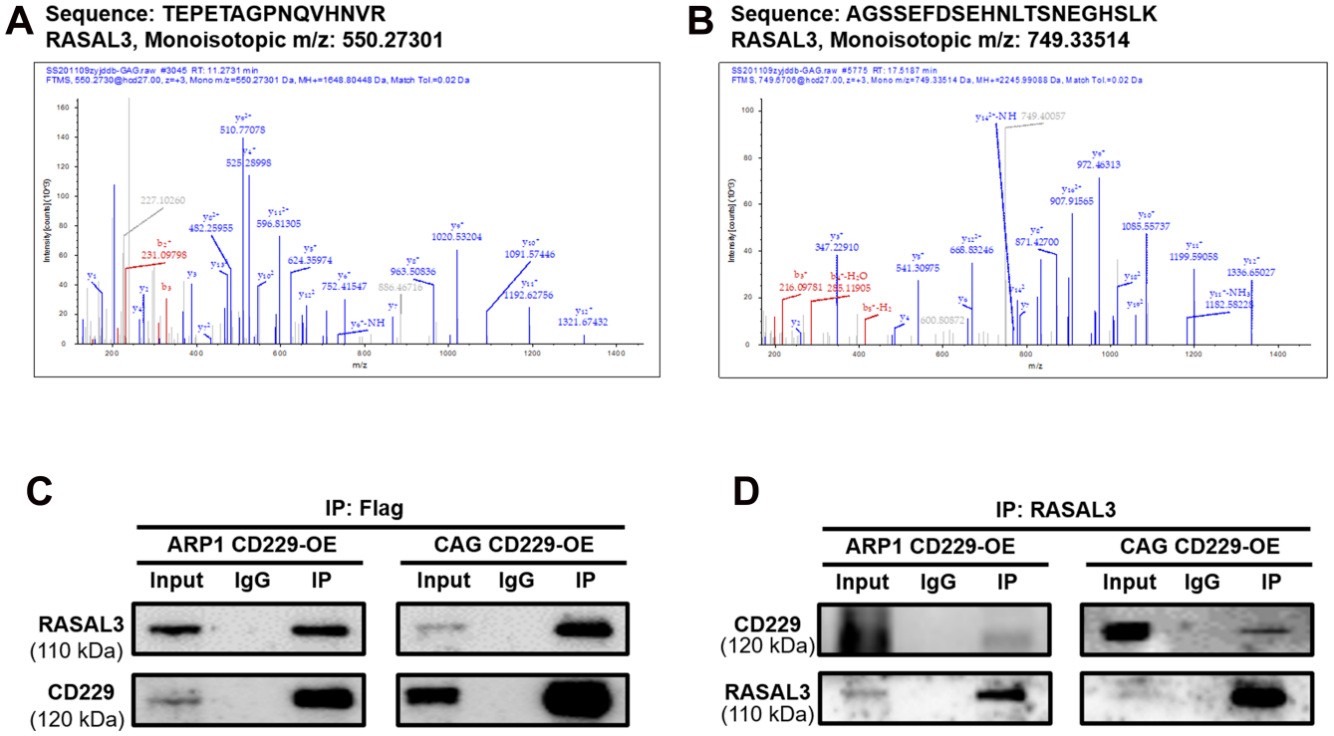
**CD229 regulates the RAS signaling pathway by interacting with RASAL3 in MM.** (**A**, **B**) The specific peptides from CD229 were identified by MS analysis. (**C**, **D**) Co-IP assay confirmed the interaction between CD229 and RASAL3. The data of experiments represent Mean±SD from at least three independent experiments.

To further explore how CD229 activated RAS, we performed Co-IP coupled with MS (Co-IP/MS) to screen the client proteins. Among these CD229-regulated proteins (see Supplementary Table 2 for Co-IP/MS data of the selected 561 proteins), two peptide fragments corresponding to a protein named RAS Protein Activator Like 3 (RASAL3) (Figure 5A, 5B) ranked the top of these client proteins. It is reported that RASAL3 contains the RasGAP domain for active RAS hydrolysis [24]. Therefore, we inferred that RASAL3 might be an important downstream protein of CD229. Co-IP experiments using FLAG antibody in CD229-OE cells confirmed the interaction between CD229 and RASAL3. Meanwhile, the IP using RASAL3 antibody could also precipitate CD229, further indicating that RASAL3 interacted with CD229 (Figure 5C, 5D). Therefore, it is reasonable to speculate that CD229 regulates the RAS/ERK signaling pathway by directly interacting with RASAL3.

### Tyrosine phosphorylation-mediated CD229 self-activation regulates the downstream RAS/ERK pathway via interacting with RASAL3

**Figure 6:**
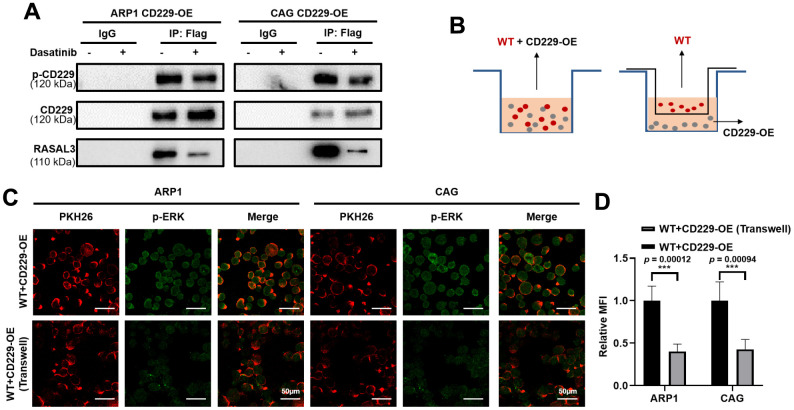
**CD229 binds to RASAL3 in a phosphorylated manner after self-activation.** (**A**) Co-IP assay detected that the phosphorylation of CD229 and the expression of RASAL3 were decreased after Dasatinib treatment in CD229-OE MM cells. (**B**) Schematic diagram of the two co-culture experiments. (**C**, **D**) Representative confocal images for PKH26 and p-ERK revealed that higher p-ERK levels were observed in the directly mixed co-culture of WT MM cells than the co-cultured cells. The data of experiments represent Mean±SD from at least three independent experiments. ***p < 0.001.

**Figure 7:**
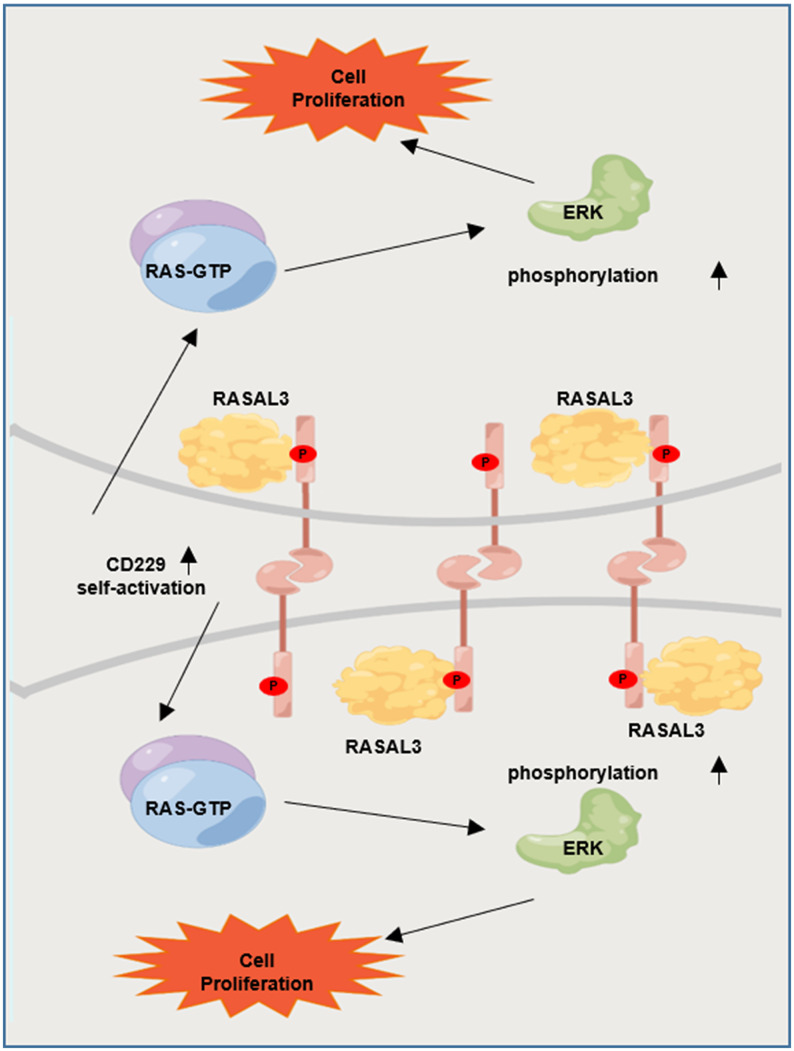
Graphic working model illustrates that tyrosine phosphorylation-mediated CD229 self-activation regulates the downstream RAS/ERK pathway by interacting with RASAL3.

It has been reported that CD229 signal transduction requires phosphorylation of the immune receptor tyrosine signal motif (ITSM) [20, 25]. We next suppressed CD229 activity using Dasatinib, a tyrosine kinase inhibitor on protein tyrosine phosphorylation [26, 27]. Co-IP experiments verified that phosphorylation of CD229 and expression of RASAL3 were blunted after Dasatinib treatment in CD229-OE MM cells, indicating the impaired interaction between CD229 and RASAL3 (Figure 6A). To further confirm the activation mode of CD229 in MM cells, we co-cultured PKH26-labeled WT MM cells with CD229-OE MM cells using transwell chambers (Figure 6B). Downstream p-ERK expression was detected by immunofluorescence assay, and higher p-ERK levels were observed in the directly mixed MM cells than the cocultured cells from transwell chambers (Figure 6C, 6D). These findings suggest that tyrosine phosphorylation-mediated CD229 self-activation activates the downstream RAS/ERK pathway by interacting with RASAL3 (Figure 7).

## DISCUSSION

MM remains a life-threatening and incurable plasma cell malignancy, while CAR-T therapy has emerged as an effective method and the curative effect from CAR-T clinical trials demonstrate a better efficacy with impressive overall response rates. Rational utilizing of targets is a prerequisite of CAR-T cell therapy. Recently, the cell surface receptor CD229 is developed as a novel and alternative CAR-T therapeutic target for MM. While the presence of CD229 on both B and T lymphocytes occurred, the CAR-T cells targeting CD229 were highly active against memory B cells and MM-propagating cells but exhibited no fratricide during CD229 CAR-T cell production. It may partially due to that the protein expression of CD229 is down-regulated in activated T cells compared to normal T cells, the CD229 CAR-T cells target normal CD229^high^ T cells during activation while spare functional CD229^neg/low^ T cells without abolishing the cytotoxicity of T cells [13, 28]. In addition, MM cells generally express high CD229 with relative specificity [29]. Our group is dedicated to the discovery of novel therapeutic targets in MM. As previously reported, we found that targeting platelet-activating factor (PAF) remodeling might be a promising strategy to enhance MM CAR-T therapy [30]. In present study, our findings further disclose that CD229 regulates MM cell proliferation through the RAS/ERK pathway and CD229 can be considered as a promising target in diagnosis and treatment of MM.

First, we verified that the elevated expression of CD229 was linked to poor prognosis in MM patients, especially in the highest risk PR subgroup with the highest degree of malignant proliferation. Our study validated that CD229 expression was significantly associated with the malignant proliferation of MM cell lines *in vitro* and *in vivo* xenograft mouse model. However, elevated CD229 expression inhibited cell proliferation, migration and induced apoptosis in hepatocellular carcinoma [31]. It is probably due to the fact that the critical role of CD229 on cell proliferation may depend on a tumor-specific background, which is important for the evolution of CAR-T.

In present study, the specific mechanism of CD229 in promoting MM cell proliferation was screened and validated by transcriptome sequencing and Co-IP/MS analysis. Among the top-ranked pathways, the enrichment correlation of endocytosis and the lysosome signaling pathway might reflect the role of CD229 as a membrane protein. Furthermore, the primary immunodeficiency signaling pathway was in accordance with the participation of CD229 in the systemic lupus erythematosus (SLE), an autoimmune disease [32]. More importantly, the RAS signaling pathway, recognized as the star cancer-related pathway, was aberrantly activated in the vast majority of cancers [33] and could lead to the malignant proliferation of cancer cells [34]. In line with previous studies, we further validated the interaction between CD229 and RAS-GTP, the active form of RAS and core indicator in RAS/ERK pathway activation [35]. We also examined the positive regulation of ERK phosphorylation in CD229-OE MM cells. However, Martin M et al. found that the interaction between TCR and CD229 resulted in partial inhibition of ERK phosphorylation after simultaneous activation of CD229 and CD3 in T lymphocytes [21], which was contradictory to our results. Since different cell receptors exist in different cells, and there is no TCR in MM cells, we infer that CD229 functions differently among multiple cell types [21]. Consistent with our results, Ishibashi M et al. reported that the ERK phosphorylation was decreased after knocking out CD229 in MM cells [36]. ERK is an important indicator of the action of RAS signaling pathway on cell proliferation [37]. In detail, for the two bands of (phosphorylated) ERK1 and ERK2 detected in Figures 3D, 3E, 4E, there was a slight difference in ERK1/ERK2 ratio between MM cell lines and *in vivo* tumor tissues, supporting their universal expression and critical regulatory role in cellular proliferation. Furthermore, RASAL3 was screened and validated as an intermediate protein between CD229 and RAS through Co-IP/MS experiment. As the most recently identified Ras GTPase activating protein, RASAL3 can accelerate RAS-GTP hydrolysis to form RAS-GDP, thereby functioning as a brake on RAS signaling pathway [38]. These results indicate that CD229 activates RAS/ERK signaling pathway by interacting with RASAL3.

CD229, as a cell surface receptor, is activated by receptor-ligand interaction [39, 40]. It is recognized that the intracellular action of CD229 depends on the tyrosine phosphorylation of its ITSM structural domain [41, 42]. Therefore, our study developed a co-culture method combined with the immunofluorescence assay to confirm the relationship between CD229 activation and downstream indicator p-ERK. We found that CD229 was self-activated followed by activating downstream pathway. Moreover, the inhibition of phosphorylation of CD229 by applying tyrosine kinase inhibitor Dasatinib reduced the interaction between CD229 and RASAL3. Therefore, it was proposed that CD229 was dependent on tyrosine phosphorylation-mediated self-activation to interact with RASAL3, thereby activating RAS/ERK signaling pathway and promoting MM cell proliferation. It is noteworthy that Dasatinib is currently a clinical agent for chronic myeloid leukemia [43] as well as relapsed MM [44]. It has been reported that Dasatinib can overcome multi-drug resistance (MDR) by inhibiting Src, increasing Bim expression and decreasing MDR1 expression in human multi-drug-resistant myeloma cells [45]. Similarly, Dasatinib can prevent MDR in RANKL-expressing MM cells [46]. Combined with the inhibitory effect of Dasatinib on CD229 activation, it is prompted that Dasatinib may be a choice in treating high-risk MM, and targeted inhibition of CD229 activation will play an adjunctive role in the treatment of MM.

In conclusion, the present study reveals that elevated CD229 expression confers poor survival in MM patients and promotes cell proliferation both *in vitro* and *in vivo*. Mechanistically, CD229 interacts with RASAL3 upon tyrosine phosphorylation-mediated self-activation, thus activating the RAS/ERK signaling pathway. Our study not only demonstrates the oncogenic role of CD229 in MM cell proliferation, but also illustrates the new theoretical basis on CD229 as a promising therapeutic target for the treatment of MM.

## MATERIALS AND METHODS

### Gene expression profiling

CD229 mRNA was determined using the gene expression profiling (GEP) cohorts, which were mined from the GEO database as previously described [47, 48]. The series matrix files were downloaded from the GEO database and imported into Excel to analyze the gene expression profiles. The optimal cutoff values were obtained by analyzing survival rates, survival status and gene expression in X-tile software. Finally, the association analysis between differentially expressed genes and patient survival was performed using the Kaplan-Meier method. GraphPad Prism 8 software was used to plot the survival curves, and the log-rank (Mantel-Cox) test method was used to test whether there was a significant difference between the high and low expression groups. The data were from GSE5900, GSE136337, Total Therapy 2 (TT2, GSE2658) and the evaluation of proteasome inhibition for extending remission (APEX, GSE9782) cohorts.

### Antibodies and reagents

The primary antibodies used in this study were at the dilutions of 1:1000 as follows: CD229 (ab103172, Abcam, UK); RASAL3 (ARP79758_P050, Aviva Systems Biology, USA); Flag (14793S, Cell Signaling Technology, USA); ERK (4695S, Cell Signaling Technology, USA); p-ERK (4370S, Cell Signaling Technology, USA); GAPDH (5174S, Cell Signaling Technology, USA). The second antibodies Goat anti-Rabbit IgG (H+L) HRP (FMS-Rb01, Fcmacs) and Goat anti-Mouse IgG (H+L) HRP (S0002, Affinity) were in 5000 diluted concentrations. Puromycin was obtained from Merck KGaA (Darmstadt, Germany). Diphenyltetrazolium Bromide (MTT) was purchased from Solarbio (Shanghai, China). PKH26 was purchased from Sigma-Aldrich (Lot#SLBP9768V, SIGMA, USA).

### Cell lines and cell culture

Human MM cell lines ARP1, CAG and peripheral blood mononuclear cells (PBMCs) were cultured in RPMI-1640 (Biological Industries, Israel). HEK293 cells were cultured in DMEM (Biological Industries, Israel). Culture medium was added with fetal bovine serum (10%, Biological Industries, Israel), penicillin (100 U/mL, HyClone, USA) and streptomycin (100 μg/mL, HyClone, USA), which was changed every 2 days. All cells were cultured in 100 mm dishes at 37° C in the 5% CO_2_ incubator.

### Plasmids and cell transfection

Plasmids containing human CD229 cDNA were provided by TranSheepBio (Shanghai, China) and CD229 siRNA were synthesized by GenePharma (Shanghai, China). The CD229 cDNA was cloned into the lentiviral vector, CD513B-1, and linked with FLAG tags. Lentiviruses containing cDNA were obtained by co-transfection of CD229 expression vector with packaging vectors (PLP1, PLP2 and PLP-VSVG) using Liposomal Transfection Reagent (Cat#40802, YEASEN, Shanghai) [49]. The virus supernatant was collected after 48 h and stored at -80° C for subsequent experiments. MM cells were transfected with lentivirus and screened by puromycin. WB test was used to verify the transduction efficiency.

The cells were resuspended with electroporation solution. Subsequently, siRNA was added into the solution to a final concentration of 100 nmol/L and then the solution was transferred into the electroporation cuvettes plus. Two pulses for 1.0 s at 960 microF of capacitance, 200 V of voltage were the most favorable electrical parameters for efficiency.

### Cell proliferation and viability assay

Cells were cultured in 96-well plates at a density of 2,000 cells/well. The Thiazolyl Blue Tetrazolium Bromide (MTT) method was performed to test the proliferation rate and cell viability for 24, 48 and 72 h, respectively. Absorbance was read at 570 nm using the microplate reader.

### Flow cytometric analysis of cell cycle

The cell cycle was detected by flow cytometry (Merck Millipore, Darmstadt, Germany) as previously described [50].

### WB and co-immunoprecipitation (Co-IP)

Protein levels were determined by WB analysis under the protocol as previously described (50). According to the manufacturer’s instructions, the Pierce Direct Magnetic IP/Co-IP kit (88828, Thermo Scientific) was utilized for Co-IP assays. As CD229 cDNA is linked with FLAG tag, the FLAG antibody was used instead of the CD229 antibody for IP. And the IgG antibody sharing the same immunogen with the IP antibody was chosen as a negative control.

### RAS activity assay

RAS activity was detected according to the instructions of the Active Ras Pull-Down and Detection Kit (16117, Thermo Scientific).

### Myeloma xenografts in NOD/SCID mice

MM xenograft model was established in 6~8-week-old SCID/NOD mice. Briefly, 1 × 10^6^ CAG CD229-OE cells were injected subcutaneously into the left abdomen of mice and the same amount of CAG WT cells were subcutaneously injected into the right abdominal cavity of mice. The diameter of the tumor was measured using a vernier caliper every 1~2 days. When the xenograft tumor diameter was up to 15 mm, the mice were sacrificed, and then the tumors were collected, weighed and photographed.

### Mass spectrometry (MS) analysis

SDS-PAGE was used to separate proteins from CD229-OE cells. Gel bands at the expected size were excised and digested with sequence-grade trypsin (Promega, USA). The proteins were first quantified, and followed by reductive alkylation to open the three-dimensional structure of the proteins. The peptides were extracted by enzymatic digestion and analyzed by MS (Q-Exactive, Thermo). Finally, the peptides were analyzed according to National Center for Biotechnology Information nonredundant protein database [48].

### Immunofluorescent staining and confocal microscopy

Immunofluorescence staining experiments were performed as previously described [51]. Images were captured using a confocal microscope (TCS SP8; Leica, Germany).

### Statistical analysis

All data were expressed as the mean ± standard deviation. Two-tailed Student’s t-test (2 groups) or one-way ANOVA for multiple comparisons were used to determine the significance between experimental groups. The Kaplan–Meier method and Log-rank test were used to determine the survival rate of MM patients. *p < 0.05 was considered statistically significant.

### Data availability statement

All supporting data are included in the manuscript and available upon reasonable request to the corresponding author. The original contributions presented in the study are publicly available. This data can be found here: the ProteomeXchange Consortium: PXD032177. The RNA-seq data was deposited in GEO (GSE199200).

## Supplementary Material

Supplementary Figure 1

Supplementary Table 1

Supplementary Table 2
