# Correction for: CD229 interacts with RASAL3 to activate RAS/ERK pathway in multiple myeloma proliferation

**DOI:** 10.18632/aging.205006

**Published:** 2023-08-28

**Authors:** Zigen Lin, Xiaozhu Tang, Yuhao Cao, Lijin Yang, Mingmei Jiang, Xinying Li, Jie Min, Bing Chen, Ye Yang, Chunyan Gu

**Affiliations:** 1 Department of Hematology, Nanjing Drum Tower Hospital, The Affiliated Hospital of Nanjing University of Chinese Medicine, Nanjing, China; 2 School of Medicine and Holistic Integrative Medicine, Nanjing University of Chinese Medicine, Nanjing, China

**Keywords:** multiple myeloma, CD229, RAS, RASAL3

**This article has been corrected:** The authors found an error in **Figure 6C**. Three images of immunostaining for PKH26, p-ERK and Merge in the co-culture transwell experiment (2nd row, 4th column, 5th column, 6th column) from the ARP1 cell line were misrepresented as the CAG cell line. The authors corrected the error with representative images of PKH26, p-ERK and Merge in CAG cells from the original experiments. Correspondingly, the quantitative results for the CAG cell line in **Figure 6D** were revised based on the replaced images. The presented corrections do not affect the results or conclusions of this article. The authors would like to apologize for any inconvenience caused.

Corrected **Figure 6** is presented below.

**Figure 6:**
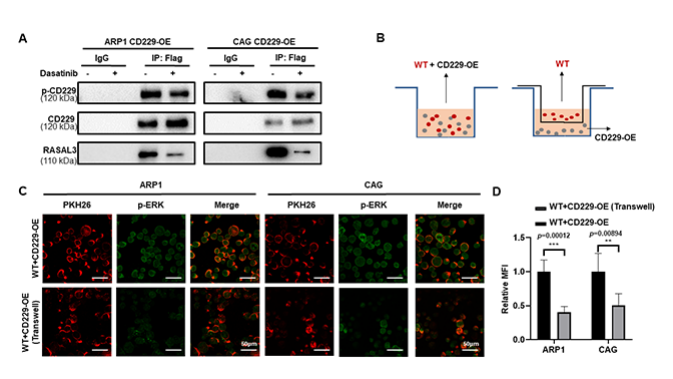
**CD229 binds to RASAL3 in a phosphorylated manner after self-activation. **(**A**) Co-IP assay detected that the phosphorylation of CD229 and the expression of RASAL3 were decreased after Dasatinib treatment in CD229-OE MM cells. (**B**) Schematic diagram of the two co-culture experiments. (**C, D**) Representative confocal images for PKH26 and p-ERK revealed that higher p-ERK levels were observed in the directly mixed co-culture of WT MM cells than the co-cultured cells. The data of experiments represent Mean±SD from at least three independent experiments. ***p < 0.001.

