# Supplementary material for: CD229 interacts with RASAL3 to activate RAS/ERK pathway in multiple myeloma proliferation
Source: Aging (Albany NY). 2022 Nov 28;14(22):9264–79. doi: 10.18632/aging.204405 (PMC9740379; doi:10.18632/aging.204405)
Supplement: Supplementary Figure 1 [file aging-14-204405-s001.pdf]

SUPPLEMENTARY FIGURE

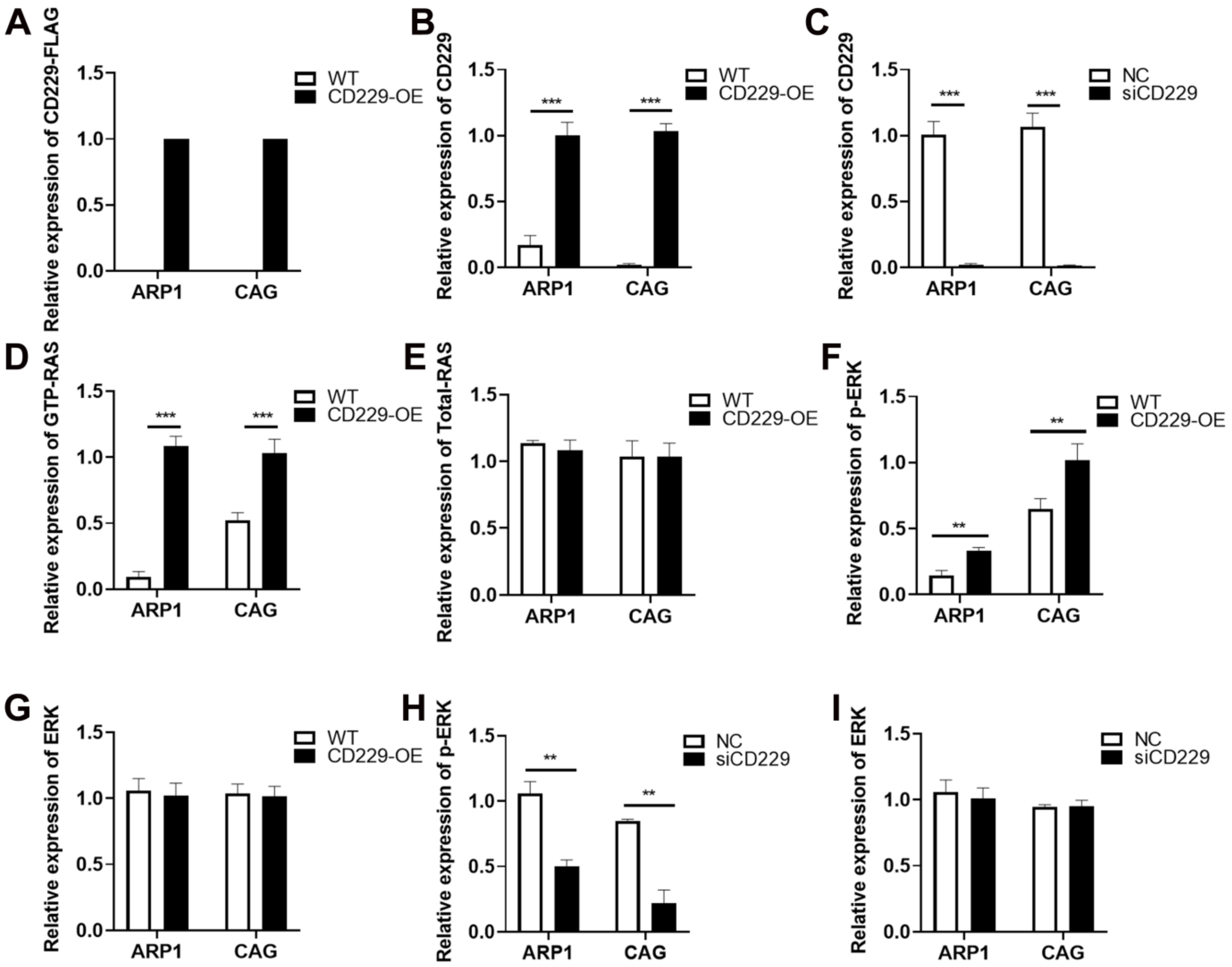

Supplementary Figure 1. The quantitative analyses of WB assays for the indicated protein. (**A**, **B**) Confirmation of CD229 expression in CD229-OE MM cells, related to Figure 2A. (**C**) Confirmation of CD229 expression in CD229-OE MM cells, related to Figure 2D. (**D**) Relative expression of RAS-GTP in WT and CD229-OE MM cells, related to Figure 3C. (**E**) Relative expression of Total-RAS in WT and CD229-OE MM cells, related to Figure 3C. (**F**, **G**) Relative expressions of p-ERK and ERK in CD229-OE MM cells, related to Figure 3D, respectively. (**H**, **I**) Relative expressions of p-ERK and ERK in siCD229 MM cells, related to Figure 3E, respectively.
